# Structural and functional alterations of intestinal flora in mice induced by halonitromethanes exposure

**DOI:** 10.3389/fmicb.2022.991818

**Published:** 2022-09-13

**Authors:** Jinbao Yin, Dingxin Li, Tianming Zheng, Xun Wang, Bin Hu, Peifang Wang

**Affiliations:** ^1^Key Laboratory of Integrated Regulation and Resources Development on Shallow Lakes of Ministry of Education, College of Environment, Hohai University, Nanjing, China; ^2^State Key Laboratory of Pollution Control and Resource Reuse, School of the Environment, Nanjing University, Nanjing, China

**Keywords:** halonitromethanes, intestinal flora, oxidative stress, inflammatory response, metabolic function

## Abstract

Halonitromethanes (HNMs) as one typical class of nitrogenous disinfection byproducts (DBPs) have been widely found in drinking water and are receiving more and more attentions because of their high cytotoxicity, genotoxicity, and developmental toxicity. However, the effects of HNMs exposure on the intestinal tract and intestinal flora remain unknown. This study comprehensively determined the effects of trichloronitromethane, bromonitromethane, and bromochloronitromethane exposure on the intestinal tract and intestinal flora. Results showed that the three HNMs induced intestinal oxidative stress and inflammatory response. Further, HNMs exposure could change the diversities and community structure of intestinal flora, thereby triggering intestinal flora dysbiosis, which might be associated with the intestinal damage such as oxidative stress and inflammation. The intestinal flora dysbiosis was accompanied with mark alterations in function of intestinal flora, such as carbohydrate, lipid, and amino acid metabolisms. This research provides a new insight into studying the toxicity of HNMs exposure based on intestinal flora, which will further improve the health risk assessment of DBPs in drinking water.

## Introduction

To prevent the spread of waterborne diseases, the disinfection is widely applied in drinking water supply to protect public health, but it inevitably produces a large number of disinfection by-products (DBPs) (Mazhar et al., 2020). More than 700 DBPs have been reported, but very few carbonaceous DBPs (C-DBPs) such as trihalomethanes (THMs) and haloacetic acids (HAAs) have been regulated due to their threat to human health (Richardson and Plewa, 2020). Accumulating evidence demonstrates that nitrogenous DBPs (N-DBPs), as typical unregulated DBPs, are more toxic than C-DBPs (Richardson et al., 2007; Zhang et al., 2022). Halonitromethanes (HNMs), as one typical class of N-DBPs, are widely detected in drinking water (0.1–3.0 μg L^−1^) and have been listed as one of the 50 high-priority monitored DBPs (Plewa et al., 2004; Hu et al., 2010). Previous studies reported that HNMs have higher cytotoxicity, genotoxicity, and developmental toxicity compared to currently regulated DBPs. Among these HNMs, trichloronitromethane (TCNM) is the first to be detected and the most frequently occurring one in drinking water, thus its toxicity has been studied. Previous studies reported that TCNM could induce high levels of DNA strand breaks in human lymphoblastoid TK6 cells and intracellular reactive oxygen species (ROS) in human epithelial cells (Liviac et al., 2009; Pesonen et al., 2015). For *in vivo* animal experiments, TCNM could react with biological thiols and inducted oxidative stress and DNA damage in mouse liver (Sparks et al., 1997; Yin et al., 2017). However, studies on other HNMs are lacking.

More than 100 trillion microbiotas are harbored in the gastrointestinal tract, which has been compared to the “invisible organ” that is essential to the human body, and plays key roles in human physiology and metabolism such as digestion and nutrition, regulation of immunity, and inflammation and oxidative stress (Zhou B. et al., 2020). Meanwhile, intestinal dysbacteriosis might cause a variety of diseases, such as obesity, metabolic disorders, and inflammatory bowel disease, even cancer (Gao et al., 2018; Khan et al., 2019; Zhou X. et al., 2020; Liu et al., 2021). It is well known that intestinal tract and intestinal flora are always affected by most DBPs in drinking water through oral ingestion. However, there are limited research data on the effects of HNMs exposure on intestinal flora.

In this study, three HNMs [TCNM, bromonitromethane (BNM), and bromochloronitromethane (BCNM)] were chosen and exposed to mice. The key biomarkers of oxidative stress and inflammation were examined in small intestine. The diversity and taxonomic composition of intestinal flora were examined by 16S rRNA gene sequencing. The functions of intestinal flora were predicted and investigated based on using Phylogenetic Investigation of Communities by Reconstruction of Unobserved States (PICRUSt, V.2.0.3-b). Results of this study could reveal the effects of HNMs on both composition and function of intestinal flora, then extend our knowledge about health risk assessment of HNMs in drinking water.

## Materials and methods

### Animal exposure

Toxicity tests were conducted on three-week-old male mice (ICR, body weight 18–22 g), which were obtained from Qinglongshan Animal Breeding Center (Nanjing, China). The mice were housed in stainless-steel cages under the ambient conditions of temperature at 25 ± 3°C, relative humidity at 50 ± 5%, light/dark cycle at 12/12 h, and food was provided *ad libitum*. After acclimated for 1 week, a total of 104 mice were randomly divided into one control group and 12 treatment groups (8 mice for each group). Control group (CK) was fed with deionized water with 0.1% dimethylsulfoxide (DMSO). The treated groups were exposed to three HNMs (TCNM, BNM, and BCNM) solution with 0.1% DMSO at four concentrations (1, 500, 5,000, and 25,000 μg L^−1^). The kinds and exposure doses of HNMs were selected according to the values of predicted LD_50_ and the mean concentrations of HNMs in drinking water, which had been explained in our previous study (Yin et al., 2017). The TCNM (purity, >99.9%), BNM (purity, >90.1%), and BCNM (purity, >93.3%) were obtained from Supelco (United States). All mice had free access to exposure water without or with HNMs, and the water was changed daily to ensure the correct concentration of HNMs.

After 30 days exposure, small intestine and fecal pellets of each mouse were collected, snap-frozen, and stored at −80°C for the following analyses. The animal experiment was administered in strict accordance with the National Institutes of Health Guide for the Care and Use of Laboratory Animals (China).

### Intestinal oxidative stress analysis

A total of 10% small intestine homogenate was prepared using phosphate buffer saline by ultrasonication. The supernatants after centrifugation were used for various measurements. As the key biomarkers of oxidative stress in small intestine, activities of superoxide dismutase (SOD), catalase (CAT), glutathione peroxidase (GSH-Px), and level of lipid peroxidation product malondialdehyde (MDA) were measured by commercial kits (Jiancheng, China). As the key biomarkers of inflammation, the levels of tumor necrosis factor-alpha (TNF-α), interleukin-1β (IL-1β), interleukin-6 (IL-6), and interferon-γ (IFN-γ) were measured by ELISA kits (Jiancheng, China). Each kit index was determined by reference to the manufacturer’s instructions. MDA levels and activities of CAT and GSH-Px were normalized by protein content. Protein contents were determined using the modified Lowry method. Each assay was run in quintuplicate.

### 16S rRNA gene sequencing of intestinal microbiota and data analyses

Total DNA from feces pellets of mice were extracted with FastDNA Soil Kit (MP Biomedicals, United States) according to the manufacturer’s instructions. The concentration and quality of the extracted DNA were determined with microspectrophotometry using NanoDrop 2000 (Thermo Fisher Scientific, United States). The V3-V4 region of bacterial 16S rRNA gene was amplified using the primer pair 341F (5′-CCTAYGGGRBGCASCAG-3′) and 806R (5′-GGACTACNNGGGTATCTAAT-3′). Individual samples were barcoded and then pooled to construct the sequencing library, then paired-end sequencing was performed on the Illumina Miseq (Illumina, United States).

The raw sequencing data were processed with Quantitative Insights into Microbial Ecology (QIIME 2, version 2021.11) for further bioinformatics analyses. Reads were quality filtered, trimmed, denoised, dereplicated, and merged using the plugin DADA2 to generate the ASV feature table. Taxonomic classification was assigned using a scikit-learn naive Bayes classifier, with a trimmed version of Greengenes 13.8 99% operational taxonomic units (OTUs). Alpha and beta diversity analyses were performed using the plugin q2-diversity in QIIME 2. Significant differences in bacteria abundance were detected by the linear discriminant analysis effect size (LEfSe).^1^ A size-effect threshold of 2 on the logarithmic LDA score was used to identify discriminating taxa.

### Bacterial metagenome content prediction

Metagenome functional content prediction was predicted from the constructed ASV feature table using PICRUSt. All predicted functional genes were annotated with Kyoto Encyclopedia of Gene and Genomes (KEGG) at different levels, and the significantly abundant pathways and generalized fold change calculations were calculated by edgeR with Fold changes ≥2.0 and FDR adjusted *p* value <0.05. UPGMA clustering of samples based on the frequencies of predicted KEGG orthologies (KOs) was performed with PAST. The z-scores of KOs were calculated with the following formula: *z*-score = (treatment abundance − control mean)/standard deviation of control (Jordan et al., 2011). A heat map was generated to visualize the functional difference among the three HNMs exposure groups using R software (version 3.6.3).

### Statistical analyses

All experimental data obtained in this study were expressed as mean ± standard deviation (SD), and One-way ANOVA test followed by Tukey’s *post hoc* test was performed for comparisons between the treatment groups and the control group using. All analyses were performed by R software (version 3.6.3). The value of *p* < 0.05 was considered statistical significance.

## Results

### Small intestine tissue damages induced by HNMs exposure

**Figure 1:**
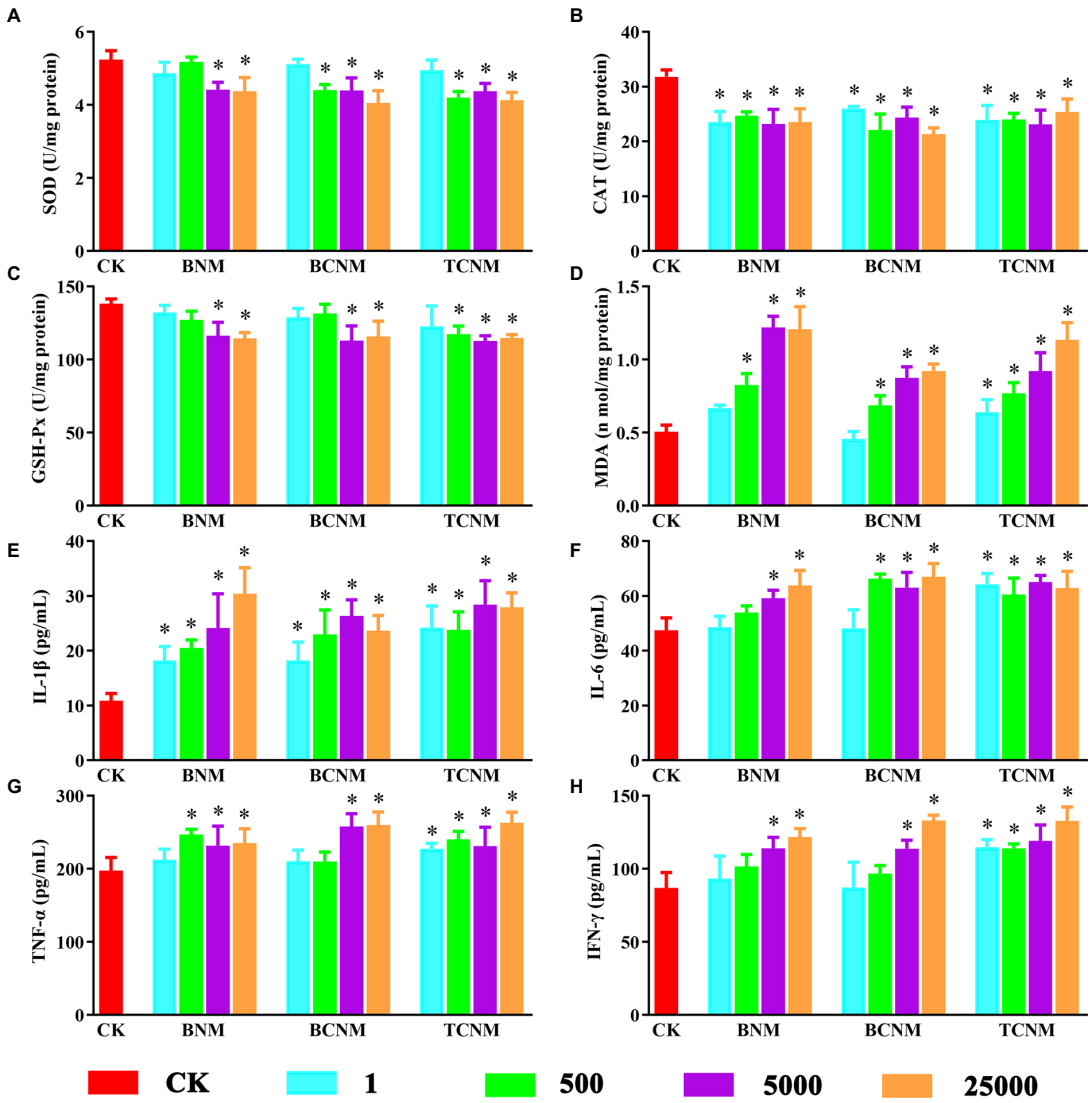
Effects of HNMs at different exposure concentrations (μg L^−1^) on the antioxidant capacity and pro-inflammatory cytokine levels in mouse small intestine. **(A)** SOD, **(B)** CAT, **(C)** GSH-Px, **(D)** MDA, **(E)** IL-1β, **(F)** IL-6, **(G)** TNF-α, and **(H)** IFN-γ. Values are mean values ± standard deviation (*n* = 5). Differences were calculated using one-way ANOVA followed by Tukey’s *post hoc* test. * means significant difference compared to CK group (*p* < 0.05).

During the whole HNMs exposure period, there was no mortality, behavioral changes, or significant difference in food and water intake for each mouse. By contrast, HNMs exposure significantly affected oxidative stress and inflammation in mouse small intestine (Figure 1). In all 5,000 and 25,000 μg L^−1^ HNMs-exposure groups, the activities of SOD and GSH-Px in mouse small intestine were significantly decreased, while the levels of MDA were increased. Meanwhile, the activities of CAT were significantly decreased in all HNMs-exposure groups. Additionally, compared to the control group, the level of IL-6, IL-1β, TNF-α, and IFN-γ were significantly increased in all 5,000 and 25,000 μg L^−1^ HNMs-exposure groups.

### Alterations of gut microbial diversity induced by HNMs exposure

**Figure 2:**
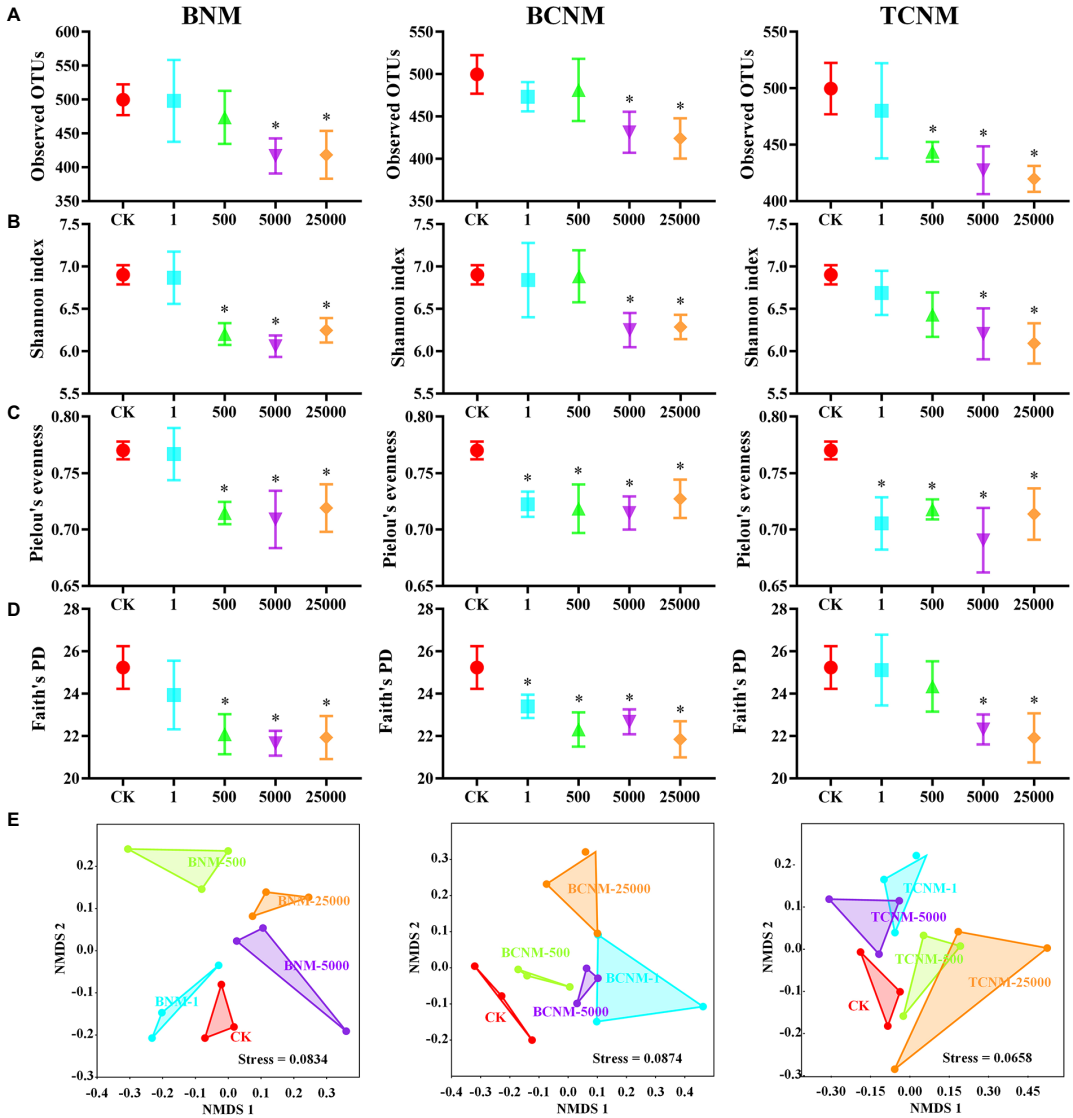
Effects of HNMs at different exposure concentrations (μg L^−1^) on the α and β-diversity of the intestinal flora in mice. **(A)** Observed OTUs, **(B)** Shannon index, **(C)** Pielou’s evenness, **(D)** Faith’s PD, and **(E)** non-metric multidimensional scaling (NMDS) based on the Bray–Curtis dissimilarity. Values are mean values ± standard deviation (*n* = 3). Differences were calculated using one-way ANOVA followed by Tukey’s *post hoc* test. * means significant difference compared to CK group (*p* < 0.05).

To investigate the intestinal flora in mice exposure to HNMs, 16S rRNA genes were analyzed by the high-throughput sequencing technique. The sequences in each sample were normalized and rarefied to 27,474 reads, and 1,612 unique AVSs were identified. The α-diversity of intestinal flora indicated by the Observed OTUs showed a dose-dependent decrease in response to HNMs exposure, and was significantly decreased in 5000 and 25,000 μg L^−1^ HNMs exposure groups (Figure 2). Similarly, Shannon index, Faith’s PD, and Pielou’s evenness showed that HNMs exposure reduced the diversity and evenness of intestinal flora in mice. In addition, β-diversities revealed by non-metric multidimensional scaling (NMDS) based on the Bray–Curtis dissimilarity showed that the intestinal flora community profiles from HNMs exposure mice were separated from those of control group, indicating a significant change of the microbial community structure induced by HNMs exposure (Figure 2).

### Alterations of intestinal flora abundance induced by HNMs exposure

**Figure 3:**
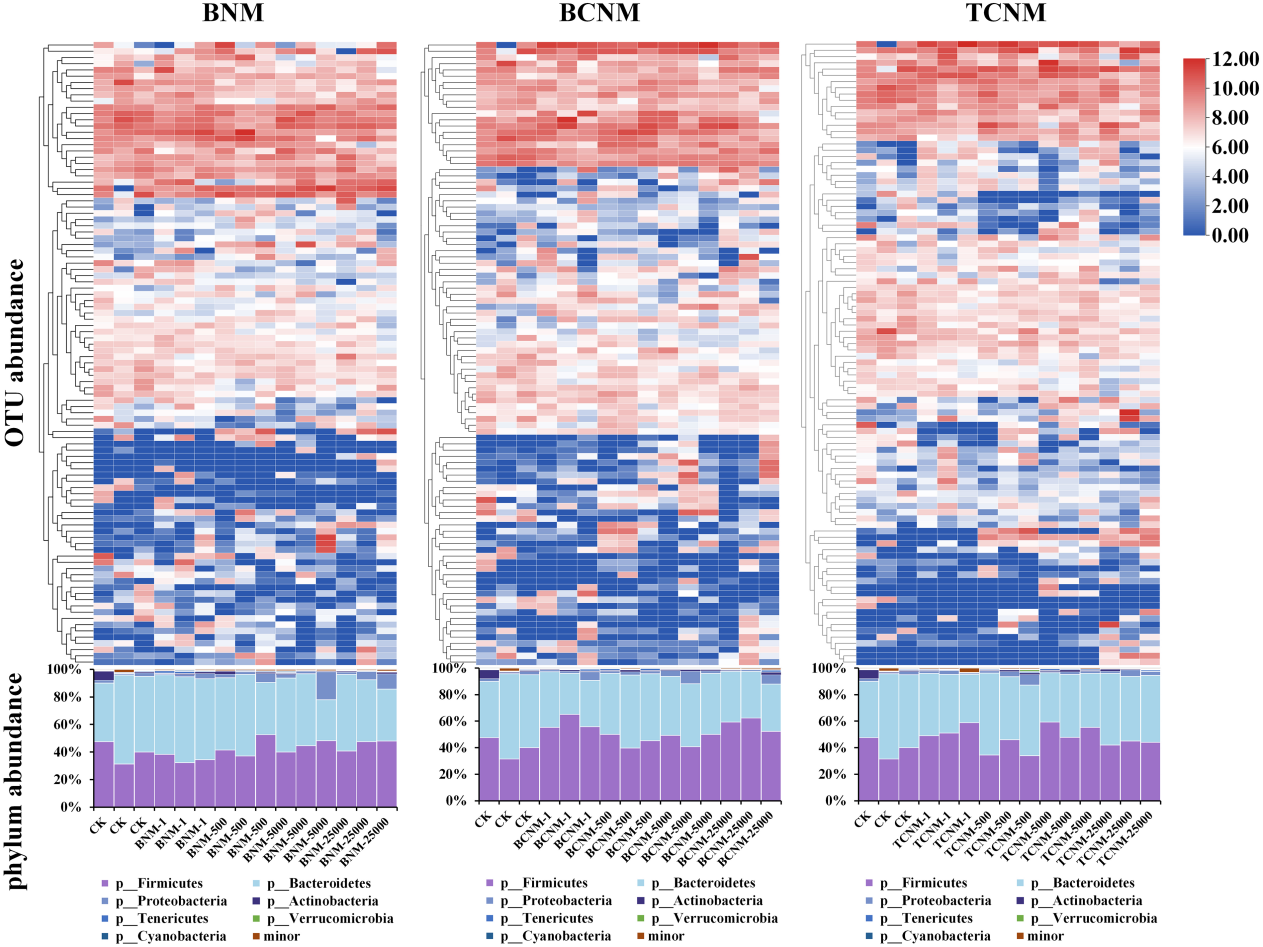
Effects of HNMs at different exposure concentrations (μg L^−1^) on intestinal flora abundance in mice. Top: Heatmap of OTUs abundance. Bottom: Stacked bar plot of relative abundances at the phylum level.

In order to clarify the effects of HNMs on the intestinal flora, taxonomic classification of intestinal flora was assigned using a scikit-learn naive Bayes classifier with QIIME2. It was found that HNMs exposure altered the abundance of many OTUs in mouse intestine (Figure 3). Meanwhile, at the phylum level, a total of 7 phyla were identified, among which Bacteroidetes (29.68–64.47%) and Firmicutes (31.37–59.48%) were the dominant phyla in all groups, followed by Proteobacteria (0.94–20.26%), Actinobacteria (0.21–6.47%), Tenericutes (0.14–1.67%), Verrucomicrobia (0–1.27%), and Cyanobacteria (0–0.88%) (Figure 3). However, the ratio of Firmicutes to Bacteroidetes (F/B ratio) was not significantly shifted by HNMs exposure (Supplementary Figure S1).

**Figure 4:**
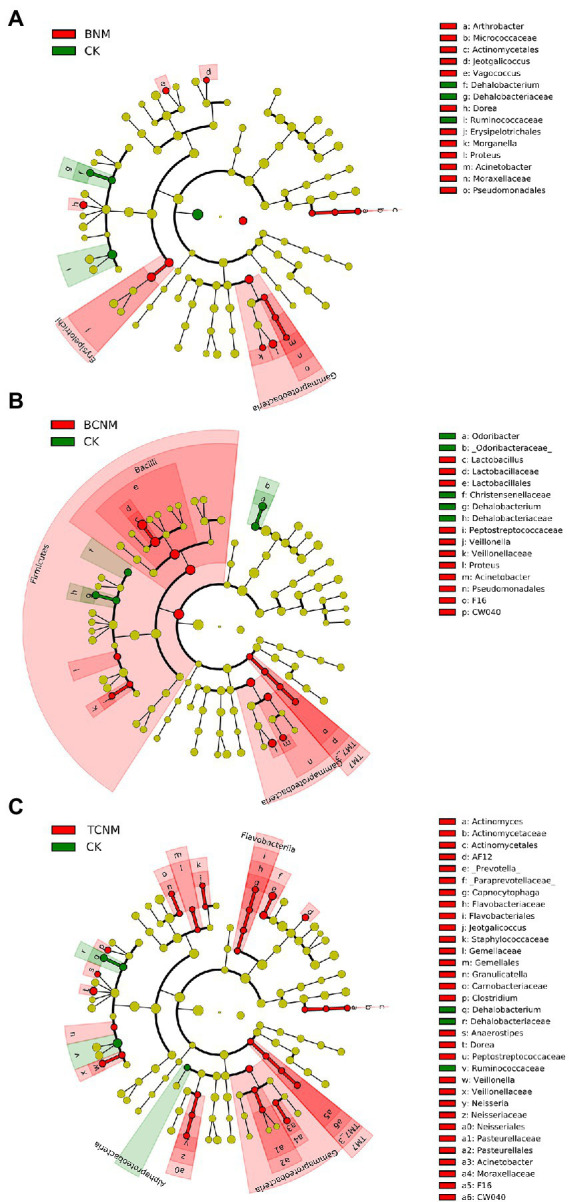
Taxonomic cladogram from LEfSe showing differences in intestinal flora by HNMs exposure. **(A)** BNM; **(B)** BCNM; **(C)** TCNM. Dot size is proportional to the abundance of the taxon. LDA score>2.

To further explore these alterations of intestinal flora abundance, we performed high-dimensional class comparisons using LEfSe, which revealed that, from phyla to genera, HNMs influenced intestinal flora in mice (Figure 4). In BNM groups, the genus *Arthrobacter, Morganella, Proteus, Acinetobacter, Jeotgalicoccus, Vagococcus,* and *Dorea* and family *Erysipelotrichales* were significantly enriched, whereas the genus *Dehalobacteriaceae* and family *Ruminococcaceae* were markedly decreased in the least one BNM exposure group when compared to the control (Figure 4A). In the least one BCNM exposure group, the genus *Lactobacillaceae, Veillonella, Proteus,* and *Acinetobacter* and family *F16* and *Peptostreptococcaceae* were more frequently observed, but the genus *Odoribacter* and *Dehalobacteriaceae* and family Christensenellaceae were less prevalent compared to that of CK group (Figure 4B). Similarly, the genus *Actinomyces, AF12, Prevotella, Capnocytophaga, Clostridium, Anaerostipes, Dorea, Veillonella, Neisseria, Acinetobacter, Jeotgalicoccus, and Granulicatella* and family *Gemellaceae, Peptostreptococcaceae,* and *F16* were more abundant, whereas the genus *Dehalobacteriaceae,* family *Ruminococcaceae,* and order *Alphaproteobacteria* were less frequently observed in the least one TCNM exposure group compared to that of CK group (Figure 4C).

### Functional changes of intestinal flora induced by HNMs exposure

**Figure 5:**
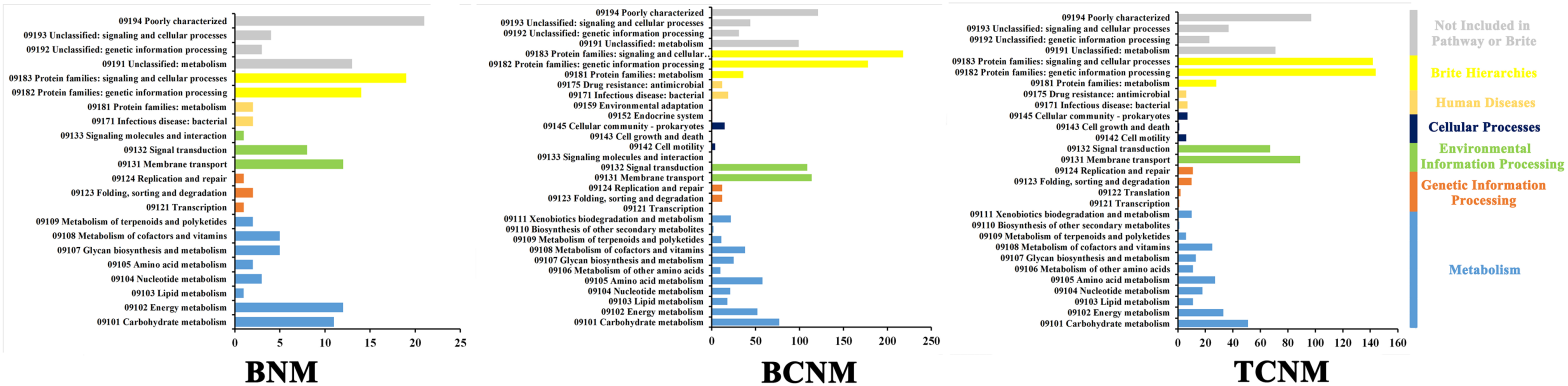
Significantly enriched KEGG pathways in intestinal flora by HNMs exposure using the PICRUST algorithm compared with the corresponding CK group.

**Figure 6:**
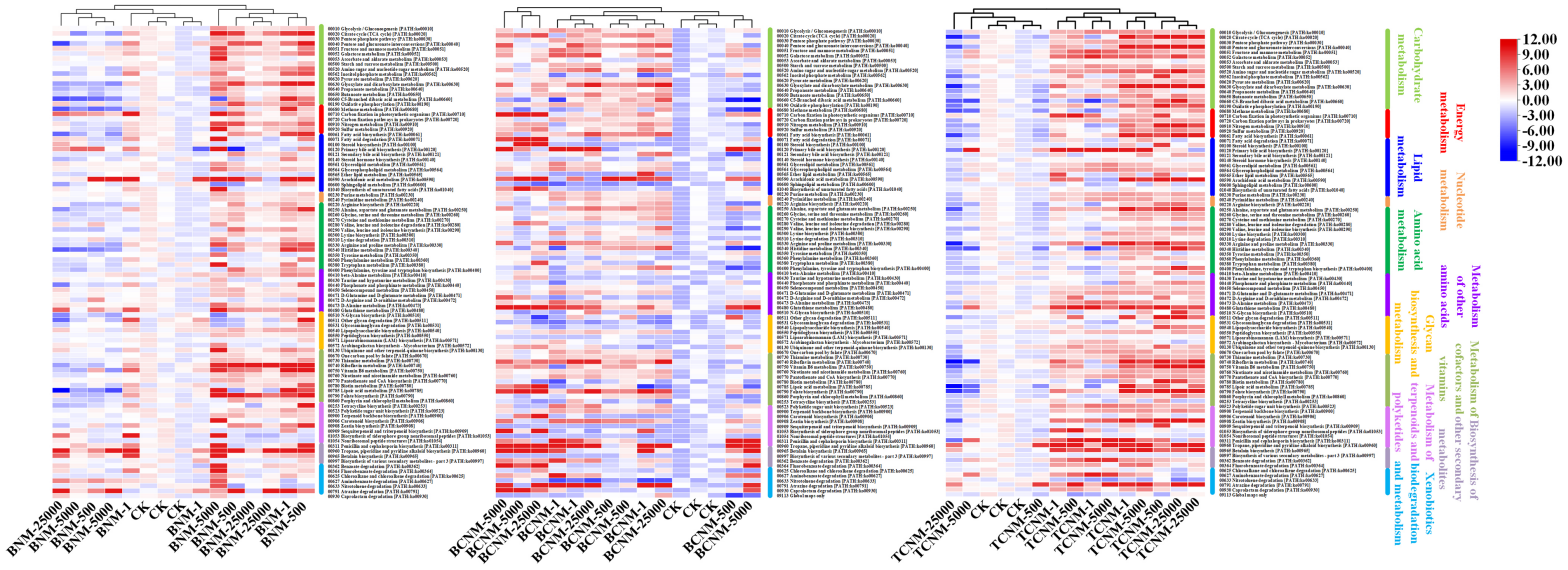
Heat map for all significantly differentially represented metabolic functional pathways in the HNMs-exposure groups calculated by z-scores.

The PICRUSt2 pipeline was used to determine the differences in functional profiles of intestinal flora in mice by HNMs exposure, and a total of 5,842 KOs were predicted. Hierarchical clustering tree showed that the HNMs exposure groups and CK group were well separated based on the frequencies of predicted KOs, which is similar to the NMDS results of intestinal flora community (Supplementary Figure S2). Differential KOs’ frequencies analysis was performed using edgeR (Fold changes ≥2.0 and FDR adjusted *p* value <0.05). 145, 1363, and 955 KOs were differentially represented in the least one BNM, BCNM, and TCNM exposure group compared to that of CK group, respectively (Supplementary Figure S3). These differential KOs were primarily related to metabolism, followed by BRITE hierarchies, environmental information processing, human diseases, cellular processes, genetic information processing, and organismal systems at KEGG level 1 (Figure 5). Notably, the processes of metabolism included energy metabolism, lipid metabolism, nucleotide metabolism, amino acid metabolism, metabolism of other amino acids, glycan biosynthesis and metabolism, metabolism of cofactors and vitamins, metabolism of terpenoids and polyketides, biosynthesis of other secondary metabolites, and xenobiotics biodegradation and metabolism. Meanwhile, most of the differential KOs in metabolism were significantly upregulated in HNMs exposure groups compared to the CK group (Figure 6).

## Discussion

Accumulating evidence strongly suggests that HNMs as one typical class of nitrogenous disinfection byproducts have higher toxicity than the regulated DBPs, such as THMs and HAAs (Plewa et al., 2004; Muellner et al., 2007). Oral ingestion of drinking water containing HNMs is the primary route of environmental exposure to HNMs. Then the intestine is directly exposed to HNMs as the main barrier after oral intake. Meanwhile, the intestine is the primary organ for nutrient metabolism and energy harvest, as well as a major site for interaction of the host-commensal microbiota (Kaiko and Stappenbeck, 2014). And intestinal flora plays an essential role in host health and diseases (Zhou B. et al., 2020). Herein, we investigated intestine injury and alterations of intestinal flora induced by 30 days exposure of BNM, BCNM, and TCNM in order to reveal the potential health risk of HNMs in drinking water.

Our recent research showed that HNMs could induce hepatotoxicity in mouse liver and oxidative stress was considered as one of the potential mechanisms of DBPs toxicity (Yin et al., 2017). Herein, the key biomarkers of oxidative stress and inflammation were to investigated reveal HNMs damages on the mouse small intestine (Figure 1). The activities of important antioxidant enzymes as SOD, GSH-Px, and CAT were significantly decreased after HNMs exposure, while the levels of MDA contents were increased. Those results indicated that HNMs exposure could inhibit the metabolism of ROS and peroxides, cause lipid peroxidation, and induce oxidative damage in small intestine. These were consistent with our previous results in mouse liver (Yin et al., 2017), as well as a previous study showed that TCNM could induce oxidative stress in the respiratory system (Pesonen and Vahakangas, 2020). Additionally, the levels of cytokines IL-6, IL-1β, TNF-α, and IFN-γ in small intestine were significantly increased after the HNMs exposure, suggesting HNMs exposure caused a small intestinal inflammatory response. A previous study has reported that many DBPs could alter the expression of genes associated with immunity and inflammation in human intestinal epithelial cells (Prochazka et al., 2019). Meanwhile, it is well known that excess ROS produced by an imbalance in the antioxidant system could cause an inflammatory response (Blaser et al., 2016). On the other hand, inflammatory response is known to generate ROS and aggravate oxidative damage through a variety of mechanisms (Snezhkina et al., 2019; Simpson and Oliver, 2020). Therefore, these findings supported our results that HNMs might damage the mouse small intestine through oxidative stress and inflammation.

Recently, several studies have suggested that the diversity and complexity of intestinal flora played an important role to maintain the host’s immune and energy metabolism (Moghadamrad et al., 2019; Rong et al., 2019). And the disorders of intestinal flora could trigger the barrier disruption and inflammatory response in small intestinal (Zheng et al., 2018; Lee et al., 2019). In this study, alterations in intestinal flora were analyzed by Illumina high-throughput sequencing. The results showed that HNMs exposure significantly decreased Observed ASVs, Shannon index, Faith’s PD, and Pielou’s evenness of intestinal flora in mice, which indicated that the diversities of intestinal flora were significantly reduced (Figure 2). Meanwhile, there was a significant difference in intestinal flora community profiles among all groups according to β-diversity by NMDS and HNMs exposure groups were separated from the control group (Figure 2). These results indicated that HNMs exposure altered the intestinal flora community construction in mice. The alterations in diversities and community structure of intestinal flora indicated that HNMs exposure could trigger intestinal flora dysbiosis, which might be associated with intestinal damage such as oxidative stress and inflammation. A previous study has reported that exposure of trichloroacetamide as an emerging nitrogenous DBP, could significantly alter the composition and function of intestinal flora in mice (Zhang et al., 2015).

In further order to clarify the effects of HNMs on the intestinal flora, taxonomic classification of intestinal flora was assigned according to the Greengenes database (Figure 3). In this study, Bacteroidetes and Firmicutes were the dominant phyla in all groups, but the Firmicutes/Bacteroidetes ratio were not significantly shifted by HNMs exposure (Supplementary Figure S1). However, the result was not consistent with the previous study that trichloroacetamide exposure significantly decreased the Firmicutes/Bacteroidetes ratio, which might due to the difference of DBPs (Zhang et al., 2015). However, the abundances of intestinal flora were significantly changed at family or genus levels (Figure 4). It was noteworthy that in BNM, BCNM, and TCNM exposure groups, *Acinetobacter* were more frequently observed. Previous studies have shown that some bacteria among *Acinetobacter*, are opportunistic pathogens causing gastroenteritis such as *Acinetobacter lwoffii* and *Acinetobacter johnsonii* (Palmisano et al., 2020). Meanwhile, *Morganella*, *Proteus*, *Veillonella*, *Actinomyces*, *Prevotella*, *Clostridium*, *Granulicatella* were significantly enriched in the least one BNM, BCNM, or TCNM exposure group, which have been proved to be closely related to gastroenteritis (Hamilton et al., 2018; Li et al., 2018; Kiu et al., 2019; Bockmeyer et al., 2020; Iljazovic et al., 2021; Tian et al., 2022; Zhan et al., 2022). Accumulating evidence strongly suggests that intestinal dysbacteriosis and enteritis-associated pathogens bloom are important causes of intestinal inflammation, lesions, and even cancer (Gao et al., 2018; Khan et al., 2019; Zhou X. et al., 2020; Liu et al., 2021). As mentioned above, it was observed inflammatory response in small intestinal, which might be caused by alteration of enteritis-associated intestinal flora exposure to HNMs. Besides, *Ruminococcaceae* and *Christensenellaceae* were markedly decreased in least one HNMs exposure group, which as important players in human health, improving gut motility or decreasing inflammation (Martoni et al., 2019; Waters and Ley, 2019). These findings suggested that HNMs exposure could disrupt the immune function of the intestinal flora and threaten human health.

Furthermore, it was investigated the functional alterations of intestinal flora induced by HNMs exposure using PICRUSt to predict metagenome functions by annotating to KEGG catalog. Hierarchical clustering tree showed alterations of predicted KOs among the HNMs exposure groups and CK group (Supplementary Figure S2), which is similar to the observations of intestinal flora community (Figure 2). Here, there was a large number of significant differential KOs primarily related to the processes of metabolism in HNMs exposure groups compared to the control, indicating that HNMs exposure markedly disturbed the metabolic pathways of intestinal flora (Figure 5). Meanwhile, most of the differential KOs associated with carbohydrate, lipid, amino acid, and glycan were significantly upregulated after HNMs exposure (Figure 6). This was verified by our previous study showing that amino acid metabolism and carbohydrate metabolism in mouse liver were disturbed by HNMs exposure by metabolomics analysis (Yin et al., 2017). Notably, the metabolites of intestinal flora, such as carbohydrate, lipid, and amino acid, induced by these disturbed metabolic pathways are important regulators of oxidative stress inflammatory response. Previous studies have demonstrated that high carbohydrate diet could induce endoplasmic reticulum stress and oxidative stress, and further promoted inflammation and apoptosis, even impaired intestinal barrier in fish and humans (Gregersen et al., 2012; Zhao et al., 2021). Lipids have been evidenced to regulate oxidative stress and cause the subsequent development of uncontrolled inflammatory responses (Mavangira and Sordillo, 2018). Interestingly, the present study revealed that MDA content levels, as lipid peroxidation, were increased in mouse small intestine exposure to HNMs. For amino acids, some amino acids have been reported to have anti-oxidative and anti-inflammatory properties (Shi et al., 2021), but a high concentration of branched-chain amino acids has properties to promote oxidative stress and inflammation in human (Zhenyukh et al., 2017). Together, these findings demonstrated that the abnormal function of intestinal flora exposure to HNMs might indirectly trigger intestinal oxidative stress and inflammatory response.

## Conclusion

This study revealed that three HNMs exposure could reduce the activities of antioxidant enzymes and increase the levels of inflammatory cytokines, suggesting that HNMs exposure induced intestinal oxidative stress and inflammatory response. Meanwhile, HNMs exposure could change the diversities and community structure of intestinal flora, thereby triggering intestinal flora dysbiosis, which might be associated with the intestinal damage such as oxidative stress and inflammation. The intestinal flora dysbiosis was accompanied with mark alterations in function of intestinal flora, such as carbohydrate, lipid, and amino acid metabolisms, which might indirectly couse intestinal oxidative stress and inflammatory response. Combined with the above results, these evidence indicate that HNMs exposure could induce intestinal oxidative stress and inflammatory response and trigger intestinal flora dysbiosis. Finally, this research provided a new insight into studying the toxicity of HNMs exposure based on intestinal flora, which would further improve the health risk assessment of DBPs in drinking water.

## Data availability statement

The datasets presented in this study can be found in online repositories. The names of the repository/repositories and accession number(s) can be found at: NCBI repository, accession number SUB11905810, BioProject PRJNA867126.

## Ethics statement

The animal study was reviewed and approved by Experimental Animal Welfare and Ethics Committee, Nanjing University.

## Author contributions

PW performed the conceptualization, writing, project administration, and supervision. JY contributed to the conceptualization, methodology, formal analysis, writing, and funding acquisition. DL, TZ, and BH carried out the data analysis and writing. All authors contributed to the article and approved the submitted version.

## Funding

This work was supported by the National Natural Science Foundation of China for Young Scholars (Grant No. 21806079), the China Postdoctoral Science Foundation (Grant No. 2021M690870), and the State Key Laboratory of Pollution Control and Resource Reuse Foundation (Grant No. PCRRF20010).

## Conflict of interest

The authors declare that the research was conducted in the absence of any commercial or financial relationships that could be construed as a potential conflict of interest.

## Publisher’s note

All claims expressed in this article are solely those of the authors and do not necessarily represent those of their affiliated organizations, or those of the publisher, the editors and the reviewers. Any product that may be evaluated in this article, or claim that may be made by its manufacturer, is not guaranteed or endorsed by the publisher.

## Footnotes

^1^ http://huttenhower.sph.harvard.edu/galaxy/

## References

[ref1] Blaser H. Dostert C. Mak T. W. Brenner D. (2016). TNF and ROS crosstal in inflammation. Trends Cell Biol. 26, 249–261. doi: 10.1016/j.tcb.2015.12.002 26791157

[ref2] Bockmeyer J. Taha-Mehlitz S. Heeren N. Ristic S. Metzger J. Gass J.-M. (2020). Jejunal diverticulosis probably leading to pylephlebitis of the superior mesenteric vein. Case Rep. Surg. 2020:2343218. doi: 10.1155/2020/2343218, PMID: 33014505 PMC7519438

[ref3] Gao X. Jia R. Xie L. Kuang L. Feng L. Wan C. (2018). A study of the correlation between obesity and intestinal flora in school-age children. Sci. Rep. 8:14511. doi: 10.1038/s41598-018-32730-6, PMID: 30267022 PMC6162261

[ref4] Gregersen S. Samocha-Bonet D. Heilbronn L. K. Campbell L. V. (2012). Inflammatory and oxidative stress responses to high-carbohydrate and high-fat meals in healthy humans. J. Nutr. Metabol. 2012:238056. doi: 10.1155/2012/238056, PMID: 22474579 PMC3306970

[ref5] Hamilton A. L. Kamm M. A. Ng S. C. Morrison M. (2018). *Proteus* spp. as putative gastrointestinal pathogens. Clin. Microbiol. Rev. 31:e00085-17. doi: 10.1128/cmr.00085-17, PMID: 29899011 PMC6056842

[ref6] Hu J. Song H. Addison J. W. Karanfil T. (2010). Halonitromethane formation potentials in drinking waters. Water Res. 44, 105–114. doi: 10.1016/j.watres.2009.09.006, PMID: 19793604

[ref7] Iljazovic A. Amend L. Galvez E. J. C. de Oliveira R. Strowig T. (2021). Modulation of inflammatory responses by gastrointestinal *Prevotella* spp. – from associations to functional studies. Int. J. Med. Microbiol. 311:151472. doi: 10.1016/j.ijmm.2021.151472, PMID: 33461110

[ref01] Jordan J. Zare A. Jackson L. J. Habibi H. R. Weljie A. M. (2011). Environmental contaminant mixtures at ambient concentrations invoke a metabolic stress response in goldfish not predicted from exposure to individual compounds alone. Proteome Res. 11, 1133–1143. 10.1021/pr200840b 22141365

[ref8] Kaiko G. E. Stappenbeck T. S. (2014). Host-microbe interactions shaping the gastrointestinal environment. Trends Immunol. 35, 538–548. doi: 10.1016/j.it.2014.08.002, PMID: 25220948 PMC4253298

[ref9] Khan I. Ullah N. Zha L. Bai Y. Khan A. Zhao T.. (2019). Alteration of gut microbiota in inflammatory bowel disease (IBD): cause or consequence? IBD treatment targeting the gut microbiome. Pathogens 8:126. doi: 10.3390/pathogens8030126, PMID: 31412603 PMC6789542

[ref10] Kiu R. Caim S. Painset A. Pickard D. Swift C. Dougan G.. (2019). Phylogenomic analysis of gastroenteritis-associated *Clostridium perfringens* in England and Wales over a 7-year period indicates distribution of clonal toxigenic strains in multiple outbreaks and extensive involvement of enterotoxin-encoding (CPE) plasmids. Microb. Genom. 5:e000297. doi: 10.1099/mgen.0.000297, PMID: 31553300 PMC6861862

[ref11] Lee S. Kirkland R. Grunewald Z. I. Sun Q. Wicker L. de La Serre C. B. (2019). Beneficial effects of non-encapsulated or encapsulated probiotic supplementation on microbiota composition, intestinal barrier functions, inflammatory profiles, and glucose tolerance in high fat fed rats. Nutrients 11:1975. doi: 10.3390/nu11091975, PMID: 31443365 PMC6769526

[ref12] Li J. Li Y. Zhou Y. Wang C. Wu B. Wan J. (2018). Actinomyces and alimentary tract diseases: a review of its biological functions and pathology. Biomed. Res. Int. 2018:3820215. doi: 10.1155/2018/3820215, PMID: 30225251 PMC6129341

[ref13] Liu J. Zhao F. Wang T. Xu Y. Qiu J. Qian Y. (2021). Host metabolic disorders induced by alterations in intestinal flora under dietary pesticide exposure. J. Agric. Food Chem. 69, 6303–6317. doi: 10.1021/acs.jafc.1c00273, PMID: 34048223

[ref14] Liviac D. Creus A. Marcos R. (2009). Genotoxicity analysis of two halonitromethanes, a novel group of disinfection by-products (DBPs), in human cells treated in vitro. Environ. Res. 109, 232–238. doi: 10.1016/j.envres.2008.12.009, PMID: 19200951

[ref15] Martoni C. J. Evans M. Chow C.-E. T. Chan L. S. Leyer G. (2019). Impact of a probiotic product on bowel habits and microbial profile in participants with functional constipation: a randomized controlled trial. J. Dig. Dis. 20, 435–446. doi: 10.1111/1751-2980.12797, PMID: 31271261 PMC6851827

[ref16] Mavangira V. Sordillo L. M. (2018). Role of lipid mediators in the regulation of oxidative stress and inflammatory responses in dairy cattle. Res. Vet. Sci. 116, 4–14. doi: 10.1016/j.rvsc.2017.08.002, PMID: 28807478

[ref17] Mazhar M. A. Khan N. A. Ahmed S. Khan A. H. Hussain A.. (2020). Chlorination disinfection by-products in municipal drinking water – a review. J. Clean. Prod. 273:123159. doi: 10.1016/j.jclepro.2020.123159

[ref18] Moghadamrad S. Hassan M. Mccoy K. D. Kirundi J. Kellmann P. Gottardi A. D. (2019). Attenuated fibrosis in specific pathogen-free microbiota in experimental cholestasis-and toxin-induced liver injury. FASEB J. 33, 12464–12476. doi: 10.1096/fj.201901113R, PMID: 31431085 PMC6902738

[ref19] Muellner M. G. Wagner E. D. Mccalla K. Richardson S. D. Woo Y. T. Plewa M. J. (2007). Haloacetonitriles vs. regulated haloacetic acids: are nitrogen-containing DBPs more toxic? Environ. Sci. Technol. 41, 645–651. doi: 10.1021/es0617441, PMID: 17310735

[ref20] Palmisano S. Campisciano G. Iacuzzo C. Bonadio L. Zucca A. Cosola D.. (2020). Role of preoperative gut microbiota on colorectal anastomotic leakage: preliminary results. Updates Surg. 72, 1013–1022. doi: 10.1007/s13304-020-00720-x, PMID: 32062786

[ref21] Pesonen M. Storvik M. Kokkola T. Rysa J. Vahakangas K. Pasanen M. (2015). Transcriptomic analysis of human primary bronchial epithelial cells after chloropicrin treatment. Chem. Res. Toxicol. 28, 1926–1935. doi: 10.1021/acs.chemrestox.5b00123, PMID: 26352163

[ref22] Pesonen M. Vahakangas K. (2020). Chloropicrin-induced toxicity in the respiratory system. Toxicol. Lett. 323, 10–18. doi: 10.1016/j.toxlet.2020.01.022, PMID: 31982502

[ref23] Plewa M. J. Wagner E. D. Jazwierska P. Richardson S. D. Chen P. H. McKague A. B. (2004). Halonitromethane drinking water disinfection byproducts: chemical characterization and mammalian cell cytotoxicity and genotoxicity. Environ. Sci. Technol. 38, 62–68. doi: 10.1021/es030477l, PMID: 14740718

[ref24] Prochazka E. Melvin S. D. Escher B. I. Plewa M. J. Leusch F. D. L. (2019). Global transcriptional analysis of nontransformed human intestinal epithelial cells (FHs 74 Int) after exposure to selected drinking water disinfection by-products. Environ. Health Perspect. 127:117006. doi: 10.1289/ehp4945, PMID: 31755747 PMC6927499

[ref25] Richardson S. D. Plewa M. J. (2020). To regulate or not to regulate? What to do with more toxic disinfection by-products? J. Environ. Chem. Eng. 8:103939. doi: 10.1016/j.jece.2020.103939

[ref26] Richardson S. D. Plewa M. J. Wagner E. D. Schoeny R. DeMarini D. M. (2007). Occurrence, genotoxicity, and carcinogenicity of regulated and emerging disinfection by-products in drinking water: a review and roadmap for research. Mutat. Res. 636, 178–242. doi: 10.1016/j.mrrev.2007.09.001, PMID: 17980649

[ref27] Rong B. Xia T. Zhang T. Feng R. Huang H. Wu Q.. (2019). Gut microbiota: a potential manipulator for host adipose tissue and energy metabolism. J. Nutr. Biochem. 64, 206–217. doi: 10.1016/j.jnutbio.2018.10.020, PMID: 30553096

[ref28] Shi C. Yue F. Shi F. Qin Q. Wang L. Wang G.. (2021). Selenium-containing amino acids protect dextran sulfate sodium-induced colitis via ameliorating oxidative stress and intestinal inflammation. J. Inflamm. Res. 14, 85–95. doi: 10.2147/jir.S288412, PMID: 33488110 PMC7814278

[ref29] Simpson D. S. A. Oliver P. L. (2020). ROS generation in microglia: understanding oxidative stress and inflammation in neurodegenerative disease. Antioxidants 9:743. doi: 10.3390/antiox9080743, PMID: 32823544 PMC7463655

[ref30] Snezhkina A. V. Kudryavtseva A. V. Kardymon O. L. Savvateeva M. V. Melnikova N. V. Krasnov G. S.. (2019). ROS generation and antioxidant defense systems in normal and malignant cells. Oxid. Med. Cell. Longev. 2019, 1–17. doi: 10.1155/2019/6175804, PMID: 31467634 PMC6701375

[ref31] Sparks S. E. Quistad G. B. Casida J. E. (1997). Chloropicrin: reactions with biological thiols and metabolism in mice. Chem. Res. Toxicol. 10, 1001–1007. doi: 10.1021/tx9700477, PMID: 9305582

[ref32] Tian Z. Zhuang X. Zhuo S. Zhu Y. Hu S. Zhao M.. (2022). Dietary inflammatory potential mediated gut microbiota and metabolite alterations in Crohn's disease: a fire-new perspective. Clin. Nutr. 41, 1260–1271. doi: 10.1016/j.clnu.2022.04.014, PMID: 35504169

[ref33] Waters J. L. Ley R. E. (2019). The human gut bacteria *Christensenellaceae* are widespread, heritable, and associated with health. BMC Biol. 17:83. doi: 10.1186/s12915-019-0699-4, PMID: 31660948 PMC6819567

[ref34] Yin J. Wu B. Zhang X.-X. Xian Q. (2017). Comparative toxicity of chloro-and bromo-nitromethanes in mice based on a metabolomic method. Chemosphere 185, 20–28. doi: 10.1016/j.chemosphere.2017.06.116, PMID: 28683333

[ref35] Zhan Z. Liu W. Pan L. Bao Y. Yan Z. Hong L. (2022). Overabundance of *Veillonella parvula* promotes intestinal inflammation by activating macrophages via LPS-TLR4 pathway. Cell Death Discov. 8:251. doi: 10.1038/s41420-022-01015-3, PMID: 35523778 PMC9076897

[ref36] Zhang D. Bond T. Pan Y. Li M. Luo J. Xiao R.. (2022). Identification, occurrence, and cytotoxicity of haloanilines: a new class of aromatic nitrogenous disinfection byproducts in chloraminated and chlorinated drinking water. Environ. Sci. Technol. 56, 4132–4141. doi: 10.1021/acs.est.1c07375, PMID: 35302737

[ref37] Zhang Y. Zhao F. Deng Y. Zhao Y. Ren H. (2015). Metagenomic and metabolomic analysis of the toxic effects of trichloroacetamide-induced gut microbiome and urine metabolome perturbations in mice. J. Proteome Res. 14, 1752–1761. doi: 10.1021/pr5011263, PMID: 25609144

[ref38] Zhao L. Liang J. Chen F. Tang X. Liao L. Liu Q.. (2021). High carbohydrate diet induced endoplasmic reticulum stress and oxidative stress, promoted inflammation and apoptosis, impaired intestinal barrier of juvenile largemouth bass (*Micropterus salmoides*). Fish Shellfish Immunol. 119, 308–317. doi: 10.1016/j.fsi.2021.10.019, PMID: 34662728

[ref39] Zheng H. You Y. Hua M. Wu P. Liu Y. Chen Z.. (2018). Chlorophyllin modulates gut microbiota and inhibits intestinal inflammation to ameliorate hepatic fibrosis in mice. Front. Physiol. 9:1671. doi: 10.3389/fphys.2018.01671, PMID: 30564133 PMC6288434

[ref40] Zhenyukh O. Civantos E. Ruiz-Ortega M. Soledad Sanchez M. Vazquez C. Peiro C.. (2017). High concentration of branched-chain amino acids promotes oxidative stress, inflammation and migration of human peripheral blood mononuclear cells via mTORC1 activation. Free Radic. Biol. Med. 104, 165–177. doi: 10.1016/j.freeradbiomed.2017.01.009, PMID: 28089725

[ref41] Zhou X. Chen C. Zhong Y. N. Zhao F. Hao Z. Xu Y.. (2020). Effect and mechanism of vitamin D on the development of colorectal cancer based on intestinal flora disorder. J. Gastroenterol. Hepatol. 35, 1023–1031. doi: 10.1111/jgh.14949, PMID: 31788852

[ref42] Zhou B. Yuan Y. Zhang S. Guo C. Li X. Li G.. (2020). Intestinal flora and disease mutually shape the regional immune system in the intestinal tract. Front. Immunol. 11:575. doi: 10.3389/fimmu.2020.00575, PMID: 32318067 PMC7147503

