# Supplementary material for: Structural and functional alterations of intestinal flora in mice induced by halonitromethanes exposure
Source: Front Microbiol. 2022 Sep 13;13:991818. doi: 10.3389/fmicb.2022.991818 (PMC9512649; doi:10.3389/fmicb.2022.991818)
Supplement: Supplementary file 1 [file Data_Sheet_1.PDF]

Supporting Information for

Structural and functional alterations of intestinal flora in mice induced by halonitromethanes exposure

Jinbao Yin[a,b], Dingxin Li[a], Tianming Zheng[a], Xun Wang[a],Bin Hu[a], Peifang Wang[a,*]

[a] Key Laboratory of Integrated Regulation and Resources Development on Shallow Lakes of Ministry of Education, College of Environment, Hohai University, 1 Xikang Road, Nanjing 210098, China

[b] State Key Laboratory of Pollution Control and Resource Reuse, School of the Environment, Nanjing University, Nanjing, 210023, China

***Corresponding author:** Peifang Wang

Email: pfwang2005@hhu.edu.cn

This Supporting Information contains:

Fig. S1 The ratio of Bacteroidetes/Firmicutes in mice from the CK and HNMs-exposure groups.

Fig. S2 Hierarchical clustering by complete linkage of Euclidean distances by KOs abundances from the CK and HNMs-exposure groups.

Fig. S3 Volcano Plot of significantly differentially represented KOs from the CK and HNMs-exposure groups. Fold changes ≥2.0 and FDR adjusted p value < 0.05.

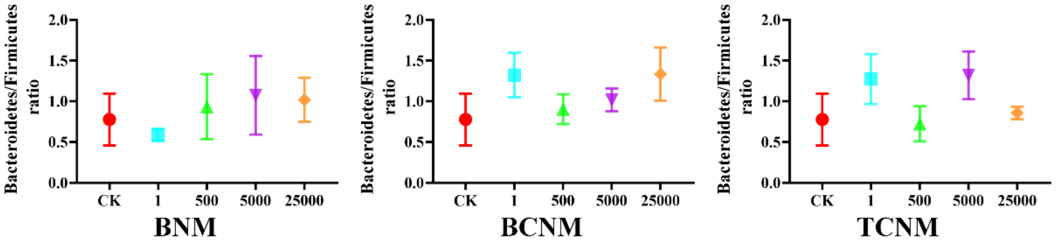

Fig. S1 The ratio of Bacteroidetes/Firmicutes in mice from the CK and HNMs-exposure groups.

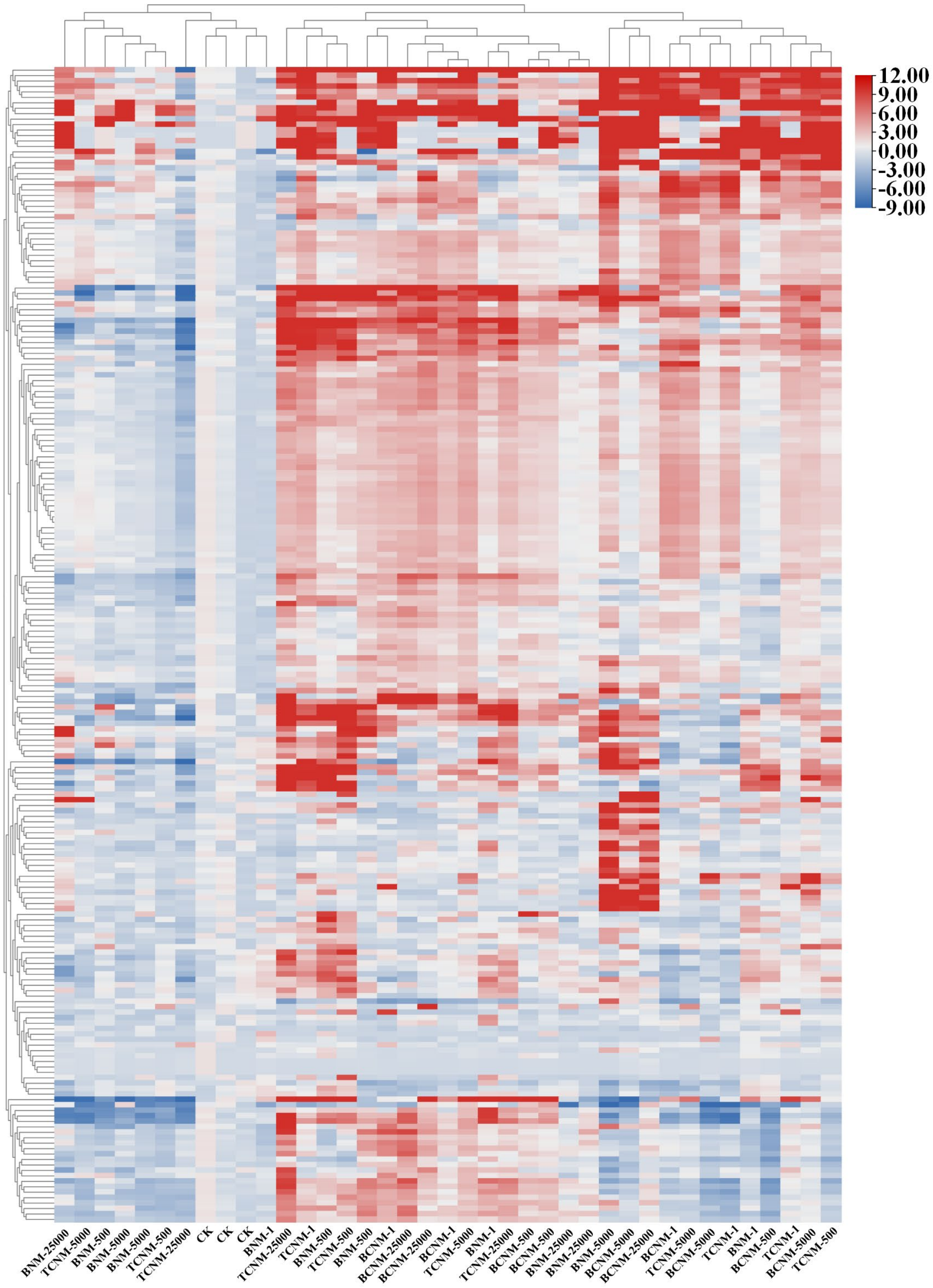

Fig. S2 Hierarchical clustering by complete linkage of Euclidean distances by KOs abundances from the CK and HNMs-exposure groups.

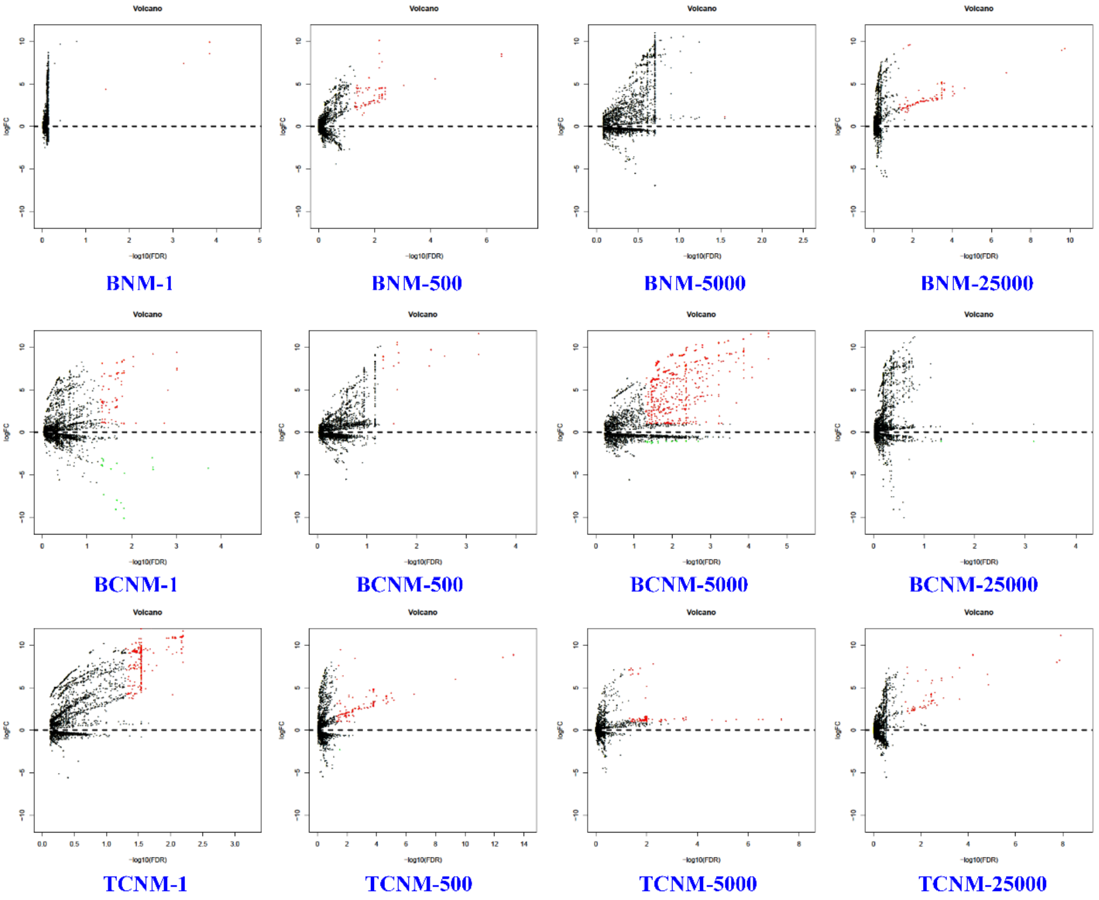

Fig. S3 Volcano Plot of significantly differentially represented KOs from the CK and HNMs-exposure groups. Fold changes ≥2.0 and FDR adjusted p value < 0.05.
